# Wearable kinesthetic system for capturing and classifying upper limb gesture in post-stroke rehabilitation

**DOI:** 10.1186/1743-0003-2-8

**Published:** 2005-03-02

**Authors:** Alessandro Tognetti, Federico Lorussi, Raphael Bartalesi, Silvana Quaglini, Mario Tesconi, Giuseppe Zupone, Danilo De Rossi

**Affiliations:** 1 Interdepartemental Research Centre "E. Piaggio", University of Pisa, Via Diotisalvi 2, Pisa, Italy; 2 Information Engineering Department, University of Pisa, Via Caruso 2, Pisa, Italy; 3 Department of Computer Engineering and Systems Science, University of Pavia, Via Ferrata 1, Pavia, Italy

## Abstract

**Background:**

Monitoring body kinematics has fundamental relevance in several biological and technical disciplines. In particular the possibility to exactly know the posture may furnish a main aid in rehabilitation topics. In the present work an innovative and unobtrusive garment able to detect the posture and the movement of the upper limb has been introduced, with particular care to its application in post stroke rehabilitation field by describing the integration of the prototype in a healthcare service.

**Methods:**

This paper deals with the design, the development and implementation of a sensing garment, from the characterization of innovative comfortable and diffuse sensors we used to the methodologies employed to gather information on the posture and movement which derive from the entire garments. Several new algorithms devoted to the signal acquisition, the treatment and posture and gesture reconstruction are introduced and tested.

**Results:**

Data obtained by means of the sensing garment are analyzed and compared with the ones recorded using a traditional movement tracking system.

**Conclusion:**

The main results treated in this work are summarized and remarked. The system was compared with a commercial movement tracking system (a set of electrogoniometers) and it performed the same accuracy in detecting upper limb postures and movements.

## Background

This work deals with the development of an innovative measuring system devoted to the analysis of the human movement. Our main aim is to provide a valid alternative comfortable instrumentation useful in several rehabilitation areas. In particular we focused our attention on the remote treatment of post-stroke patients [1].

The analysis of human movement is generally performed by measuring kinematic variables of anatomic segments by employing accelerometers, electrogoniometers, electromagnetic sensors or cameras integrated in finer equipment as stereophotogrammetric systems. In remote rehabilitation tasks, several disadvantages derive from the use of these technologies, which are mainly applied in the realization of robotics or mechatronics machines (such as MIME or MIT-MANUS [2]) which result invasive, complex and often unable to satisfy safety requirements for the presence of mechanical parts in movement. In literature, several studies are devoted to realize electric devices with properties of hight wearability [3-5]. The main drawbacks of wearable sensing systems available on the market are their weight, the rigidity of the fabric which they are made of, the dimension of the sensors used, and all the other properties which make them obtrusive. In particular, conventional sensors often require the application of complex and uncomfortable mechanical plug in order to position the sensors on garments. In the present work, we focused our efforts in the realization of a new system for the measurement of the human upper limb kinematic variables based on a sensorized garment, the Upper Limb Kinesthetic Garment (ULKG). Lightness, adherence and elasticity have been privileged in the ULKG realization as fundamental requirements for its unobtrusivity. These guidelines have led us to choose an elastic fabric (Lycra) to manufacture it as a sensorized shirt. In order to equip the lycra shirt with a sensing apparatus, sensors have been spread on the fabric by employing an electrically conductive elastomer (CE). CE deposition does not change the mechanical characteristics of the fabric. It preserves the wearability of the ULKG and it confers to the fabric piezoresistive properties related to mechanical solicitations. This property has been exploited to realize many other sensorized garments as gloves, leotards, seat covers capable of reconstructing and monitoring body shape, posture and gesture [6]. Furthermore, by using this technology, both sensors and interconnection wires can be smeared by using the same material in a single printing and manufacturing process. This is a real improvement in terms of comfort performed by the device because no metallic wires are necessary to interconnect sensors or to connect them to the electronic acquisition unit. In this way no rigid constraints are present and movements are unbounded.

## Methods

### Materials

CE composites show piezoresistive properties when a deformation is applied and can be integrated into fabric or other flexible substrate to be employed as strain sensors. Integrated CE sensors obtained in this way may be used in posture and movement analysis by realizing wearable kinesthetic interfaces [7]. The CE we used is a commercial product by WACKER Ltd (Elastosil LR 3162 A/B) [8] and it consists in a mixture containing graphite and silicon rubber. WACKER Ltd guarantees the non-toxicity of the product that, after the vulcanization, can be employed in medical and pharmaceutical applications.

### Kinesthetic Wearable Sensors

In the production process of the ULKG, a solution of Elastosil and trichloroethylene is smeared on a lycra substrate previously covered by an adhesive mask. The mask has been designed according to the desired topology of the sensor network and cut by a laser milling machine. After the CE deposition, the mask is removed and the treated fabric is placed in an oven at a temperature of 130°C to speed up the cross-linking process of the mixture. In about 10 minutes the sensing fabric is ready to be employed to manufacture the ULKG.

### Sensor Characterization

The main aim of the CE sensor characterization has been the determination of the relation between the electrical resistance *R*(*t*) of a treated fabric sample and its actual length *l*(*t*). Moreover, an analysis of the thermal transduction properties and aging of the fabric has been executed [5].

In terms of quasi-static characterization, a sample of 5 mm width shows an unstretched electrical resistance of about 1 kΩ per cm, and its gauge factor (GF) is about 2.8 , where *R *is the electrical resistance, *l *is the actual length, *R*_0 _is the electrical resistance corresponding to *l*_0 _which represents the rest length of the specimen). The temperature coefficient ratio is 0.08 K^-1^. Capacity effects showed by the sample are negligible up to 100 MHz.

### Dynamic Characterization

Electrical resistance behavior of the examined CE samples during a deformation has been fundamental to allow us to employ them as sensors. Two different issues had to be addressed to use CE as strain sensors. The first one concerns the length of the transient time, which can take up to several minutes. It is obvious that these physical systems cannot describe human movement without a signal processing devoted to compensate the slowness of this phenomenon. Moreover, electrical trend of the analyzed specimen shows some non linear phenomena which are not negligible under certain working conditions, in particular when fast deformations are applied. In this work the following results will be introduced. The typical electrical behavior of this system, when deformations in length are applied, will be described. The results of our study will lead to the formulation of a mathematical model which approximates the sensor electrical behavior. This model will be used to implement an algorithm devoted to the system regulation which consents the sensor length determination in real time. Finally, two simplified and faster versions of this sensor length determination technique will be presented and applied in posture reconstruction.

**Figure 1:**
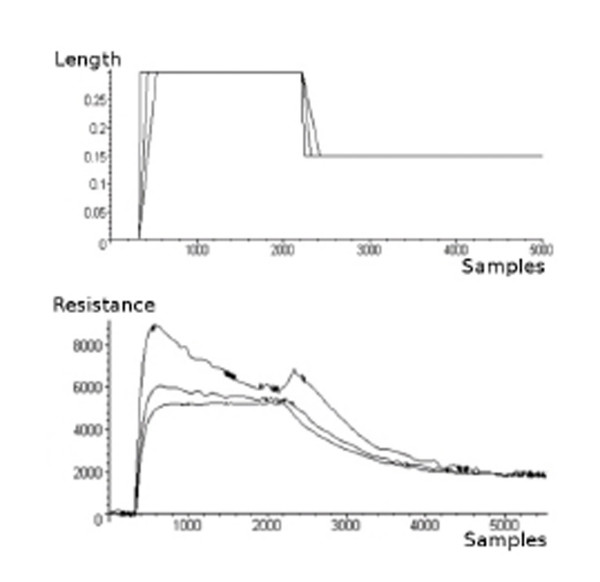
Response of a CE sensor solicited by trapezoidal ramps in deformation.

The analysis of the electrical trend of CE sensors, when deformations are applied, has been performed by using a system realized in our laboratories which can provide controlled deformations and at the same time can acquire the resistance value performed by the specimen. A wide description of this instrumentation and its performances can be found in [5]. By using this device, several deformations, which differ in their forms versus time, amplitudes and velocities have been applied to CE specimens. Figure 1, which has been reported as an example of this analysis, shows the output of a sample stretched with trapezoidal ramps in deformation having different velocities (*t*) (where *l*(*t*) is the length of the sample). The main remarks on sensor electrical behavior are summarized in the following:

• Both in case of deformations which increase the length of the specimen and in case of de formations which reduce it, two local maxima greater than both the starting value and the regime value are performed.

• If the relationship between *R*(*t*) and *l*(*t*) were linear, one of the extrema described in the previous point would be a minimum.

• The height of the overshoot peaks increases with the strength velocity ((*t*)).

• The relaxing transient time, which lasts up to several minutes, is too long to suitably code human movement.

Nonlinearity in the functional which relates *R*(*t*) and *l*(*t*) suggested us to choose an approximation containing a quadratic term in the strain velocity ((*t*)). Let us consider:



where *a*_1_, *a*_2 _and *a*_3 _are three nonzero real numbers. By using experimental data, we have verified that when the specimen is motionless, i.e. (*t*) = 0, the signal deriving from the sensor is representable by a linear combination of exponential function:



and the values *ω*_*i *_do not depend on the amplitude and velocity for a wide range of the solicitation previously applied (0 – 50 per cent of the rest length and 0 – 0.1 m/s), but they vary only according to the shape and the dimensions of the specimen and on the percentages of the components in the mixture used to realize it [9]. By considering *g*(*t*) as the input function of the differential linear system



where , we have obtained encouraging results in signal modelling [9]. In particular we have approximated the sensor behavior as the solution of a second order linear system based on equation (3):



with



where *ω*_1 _and *ω*_2 _are the two poles of the linear system (4). This relation provides an obvious (almost theoretically) method to calculate *g*(*t*). Since equation (3) contains only *R*(*t*) and its derivatives, it s simple to determine the value of *g*(*t*). So to obtain *l*(*t*)in real time it is necessary to integrate the differential equation (1) (in which the three parameters *a*_1_, *a*_2 _and *a*_3 _have been identified through the values of peaks excursions in the responses of the sensor). Unfortunately, equation (1) is not generally integrable when *g*(*t*) is unknown and its solution *l*(*t*) has to be numerically computed. This is not a simple issue because the acquired data are affected by noise and sample errors. Good results have been obtained off-line by using a wide digital filtering which used the average value of a large number of sample to reduce the noise, but introduced a signal delay [9]. Next developments will be aimed at implementing the length detection in real time during a motion.

Conversely, the problem has been already addressed when the system is motionless, i.e. (*t*) = 0 and *g*(*t*) = *a*_1_*l*(*t*), and will be treated in the next section.

### Transient Time Reduction

After a mechanical solicitation, CE sensor resistance changes according to equation (2). Unfortunately, the values determined for *ω*_*i *_and the resulting transient time do not allow to directly employ the acquired signals for our applications. On the other hand, by using equation (2) it has been possible to regulate the sensor response by calculating the coefficients *c*_*i *_(and in particular *c*_0_, which represents the final value of the signal) early with respect to the transient time duration. Since the pole values are invariant with the deformation, in order to apply relation 2, they have to be calculated only once, during the system parameter identification. If the *ω*_*i *_are known only the *c*_*i *_remain undetermined and have to be computed in real time after each deformation. The parameter identification is realized by an utility package which performs a minimization of the quantity



over a lattice *L *which spans the variables *c*_0 _... *c*_*p*_, *ω*_1 _... *ω*_*p *_and where **y **is a k-dimensional vector containing the acquired data during the transient time after a solicitation. The choice of *k *is due to the noise which affects the signal. The precision in the parameters identification increases with its value. Practically this procedures is repeated several times and the values obtained for the *ω*_*i *_are the average response evaluated on all the trials. When we have determined the pole values, after each solicitation coefficients *c*_0 _... *c*_*p *_have to be re-calculated to return the steady-state response and the related sensor length. We have developed two different procedures to calculate them. The first one consists in considering the iterate p derivatives of function (2) with respect to *t*. If *k *≥ *p*, the set of these equations evaluated on *k *samples and compared with the numerical derivatives of the signal stored in vector y constitutes a welldimensioned linear system in the variables *c*_*i*_, which can be calculated with low computational cost. Although this methodology is clear and elegant, it presents a serious disadvantage. The computation of the numerical derivatives of the signal **y **is corrupted by the noise which affects the signal. Moreover the sampling noise due to the analog-digital converter in the electronic acquisition system is amplified by its derivation. Practically, this strategy is inapplicable in this form. Results remarkably improve if analogical derivators are used. This solution addresses the problems introduced by the noise, but dramatically increases the dimension of the electronic acquisition system, because in addition to the derivators, each signal and its derivatives have to be individually acquired, and the number of the acquisition channels increases according to derivative order we use [10].

**Figure 2:**
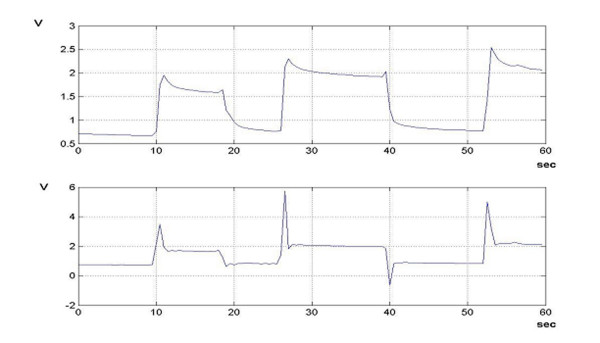
Output of a CE sensor (Voltage vs. Time) for three different deformation steps imposed (above) and treated signal (below). The transient time has been reduced.

To address this issue and attenuate noise components due to the coupling between high impedence front-ends to the connecting wires embedded in the garment and powerlines [10], we developed an algorithm based on iterative integrations of equation (2). Coefficients {*c*_*i*_}_*i *= 0...*p *_are in this case the solution of an over-dimensioned linear system *n *× *p*, obtained by integrating *n *times equation (2) on the interval [*t*_0_, *t*_*k*_]. It is trivial to prove that the obtained system is consistent for *n *≥ *p *and *k *≥ *p *by computing the jacobian matrix of the system in its parametrical form. The choice of *n *>*p *produces a filtering (based on a least square evaluation of redundant data) of signals while the coefficients are calculated. A further stabilization is due to the integration on all the interval where eq. (2) holds, by collecting all the information previously stored. No particular disadvantages arise from this methods. All the calculation is digitally computed with neither increasing the dimension of the electronic acquisition system nor introducing or amplifying further noise. The main shortcoming of this approach is that it requires that one detects each movement because equation (2) holds when the specimen is motionless, only, and the numerical integration has to be reset after each solicitation. Results are reported in Figure 2

### Realization of the Upper Limb Kinesthetic Garment

The sensing fabrics described above can be employed to realize wearable sensing systems able to record human posture and gesture, which can be worn for a long time with no discomfort. In order to realize the ULKG, we have integrated sensors into a shirt connected to an electronic unit which operates a pre-filtering process. The very innovative goal we obtained consists in printing the set of sensors and the connecting wires directly on the fabric by using CEs (in the earlier prototypes the interconnections were realized by means of metallic wires [5], which might bound movements and create artifacts). In order to realize a sensorized shirt able to monitor the kinematics of the upper limb, we have to determine position and orientation of sensors attached to the considered joints. A crucial point here is based on the observation that a redundant number of sensors (i.e. a number of sensors bigger than the number of the degrees of freedom to of the system under consideration) distributed on a surface can provide enough information to infer the essential features concerning the posture of a subject, neglecting the precise sensor location. We borrow this approach from biological paradigms [6,7]. A theoretical approach has been tried, by searching an optimization criterion to maximize the global content of information collected by the sensor system [11]. Unfortunately, this technique is very onerous in terms of required computational resources. The optimization of this calculation is at the present under study. Finally, an heuristic approach has been adopted. By realizing a sample of sensorized fabric and by placing it around the considered joints during the execution of natural movements we have determined the set of position which produces meaningful outputs in terms of movement reconstruction.

### ULKG Electrical Model and Electronic Implementation of the Acquisition Technique

**Figure 3:**
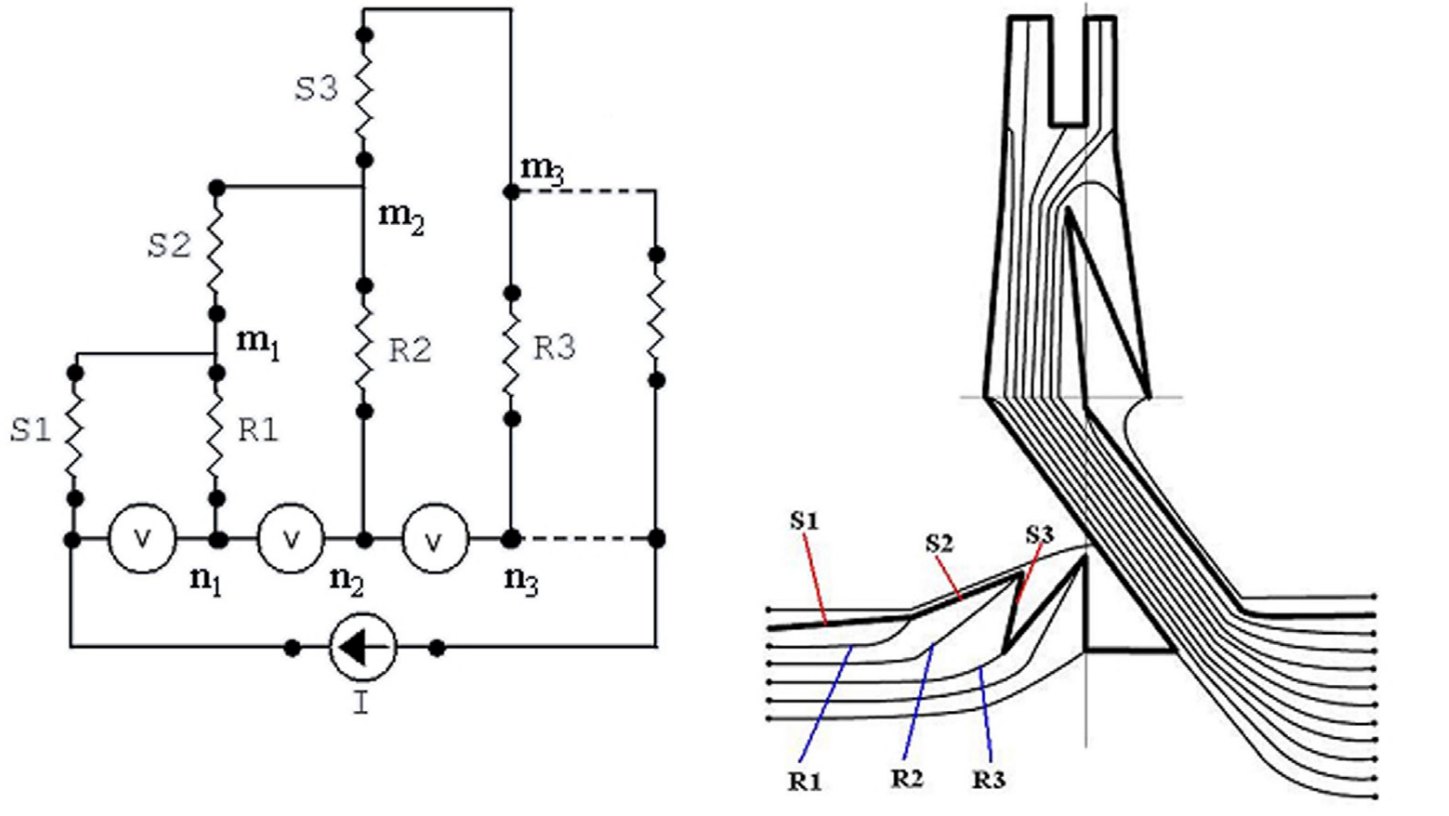
The electronic acquisition scheme (on the left) and the mask utilized for the realization of the ULKG (on the right).

**Figure 4:**
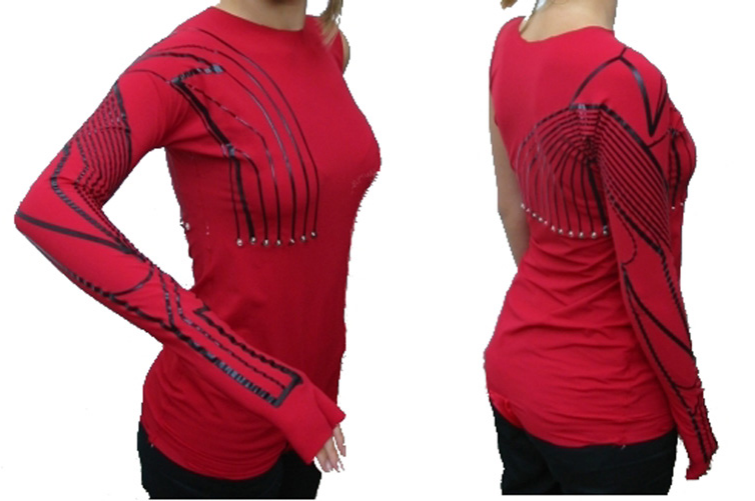
The UKLG prototype.

All the remarks and trials exposed in the previous section lead us to design the adhesive mask used to smear sensors and wires reported in Figure 3. The sensorized prototype shirt, realized by using this mask, is showed in Figure 4. The bold black track of Figure 3 represents the set of sensors connected in series (*S*_*i*_, and covers the joints of the upper limb (shoulder, elbow and wrist). The thin tracks (*R*_*i*_, Figure 3) represent the connection between the sensors set and the electronic acquisition system. Since the thin tracks are made of the same piezorestive CE mixture, they undergo a not negligible (and unknown) change in their resistance when the upper limb moves. Therefore the analog front-end of the electronic unit is designed to compensate the resistance variation of the thin tracks during the deformations of the fabric. The electric scheme is shown in figure 3. While a generator supplies the series of sensors *S*_*i *_with a constant current I, the acquisition system is provided by an high input impedance stage realized by instrumentation amplifiers and represented in Figure 3 by the set of voltmeters. Thanks to this configuration, only a little amount of current flows through the connecting wires, which have resistance values *R*_*i*_, and so the voltages which fall on *R*_*i *_are negligible if the current I, which flows in the series of sensors, is big enough. In conclusion, the voltages measured by the instrumentation amplifiers are equal to the voltages which fall on the *S*_*i *_that is related to the resistances of the sensors. In this way, the thin tracks perfectly substitute the traditional metallic wires and a sensor, consisting in a segment of the bold track between two thin tracks, can be smeared in any position to detect the movements of a certain joint.

### The ULKG Working Modes: Reconstruction of Kinematic Configurations

In order to clarify how posture detection can be done by using a kinesthetic garment, some remarks are necessary. First, in order to formally define a posture, it is necessary to develop a geometrical model of the kinematic chain under study. This can be easily done by fixing a certain number of cartesian frames, one for each degree of freedom considered and relating them with the segments which compound the kinematic chain. A kinematic configuration consists in the set of the mutual positions of the cartesian frames. Obviously, the entire set of the mutual positions is not necessary to reconstruct a posture exactly, and a minimal set can be chosen in many different ways. The Denavit-Hartemberg formalism [12] is an example of a method which fixes the exact number of relations between frames and gives a standard method to write their positions in terms of rotation and translation affinities, for rotational and translational joints.

When the ULKG is worn by a user which holds a given position described by the geometrical model, the set of sensors assumes a value strictly related to it. If the number of sensors is large enough and if the sensor locations are adequate, the values presented by them uniquely characterize the considered position. Let  be the sensor space, i.e. the vectorial space whose elements contain the values presented by sensors and where k is equal to the number of sensors in the ULKG and let  be the space containing the kinematic configurations, i.e. the space of the lagrangian coordinates that define mutual segment positions in an upper limb kinematic model, where n is equal to the number of degrees of freedom considered. To execute a reconstruction of the kinematic configuration, by knowing the sensor status, a function *F *which maps **S **into **Θ **has to be defined. We have implemented F both by a clusterization of the space **S **via a clustering norm technique into the space Θ and by the interpolation of the discrete map produced by the clusterization. In the present application the first solution has been applied by using the norm



as a clustering function, where  ∈ **S **is a *k*-dimensional vector which represents a center of the clusterization lattice and *s *∈ **S **is a *k*-dimensional vector representing the real values assumed by the sensors. Each points s* whose distance from a certain point of the lattice * is less than a previously fixed threshold *ε *is related to the value that the map assumes in *. The values which the function holds in the points of the clusterization lattice is experimentally acquired. The other implementation of *F *is described in [7] and will be summarized in section The ULKG as Posture Detector.

### Kinematic Models of Human Joints – The Upper Limb Model

In many disciplines as biomechanics, robotics and computer graphics, geometric hierarchical structures are used in articulated body modeling for robots, human or other creatures representations. An articulated body can be thought as a series of rigid segments connected by joints. A biological kinematic chain is exactly an articulated body. In the present work we implement an upper limb kinematic model by employing ideal joints in order to maintain a practical parameterization of movements without trivializing human motion. From a macroscopic point of view, a complete upper limb model would have at least 7 DOFs, corresponding to rotational movements. These ones, described by kinesiology [13], are reported in Table 1. In the model we have developed, the gleno-humeral joint of the shoulder has been parameterized as a ball and socket joint, whereas elbow and wrist consist in two successions of two rotational joints. This choice has been made in order to have an intuitive kinematic reconstruction in terms of practical mathematical characterization. Three different parameterization techniques are usually considered to describe orientations between frames:

**Table 1:** Upper limb model DOFs

shoulder	elbow	wrist
flexion-extension abduction- adduction intra-extra rotation	flexion-extension pronation-supination	flexion-extension abduction- adduct ion

• the Euler's angles;

• the exponential map;

• the unit quaternion representation.

There is not a general criterion to prefer one parameterization with respect to the others. The choice depends on the particular application; however, a good comparison can be found in [14]. The crucial point, as a classic control problem, is the presence of singularities. Euler's angles describe the orientation of a cartesian frame with respect to another by using three parameters, but have two singularities, known as gimbal-lock [15]. The exponential map introduces a new parameter with respect to Euler's angles but solves only one singularity. To address both the singularities, unitary quaternions can be used. The set of quaternions  is a non-commutative algebra of iper-complex numbers created in 1843 by Sir R. Hamilton. The unitary quaternions constitute a subgroup in  of the quaternions which have unitary cartesian norm. A clear summary of their geometric properties as vectors and their algebra can be found in [16]. We have developed our model by using both Euler's angles and unitary quaternions. This choice is due to the simplicity of the first parametrization which allows to calculate posture with low computational cost, and the necessity to realize graphic animations which interpret human movements. In [16] a methodology capable to perform fluid and biomimetic movements by using unitary quaternions is explained. We have applied Shoemake's results to represent the transition of our geometrical model and to animate an avatar piloted by the signals recorded by the ULKG.

### The ULKG as Posture and Movements Recorder

Using the ULKG, it is possible to detect if two postures are the same or not with a certain tolerance, and it is possible to record a certain set of postures coded by the status of the sensors. In the same way, movements can be recorded as transitions from one posture to another, and they are coded by the evolution of the sensor values. In particular, we have tested this capability on a set of functional relevant postures. The ULKG showed good capabilities of repeatability, even if it is removed and re-worn. An ad-hoc software devoted to recognize recorded postures has been developed. The software is able to:

• record a set of defined postures of the upper limb in a calibration phase,

• recognize the recorded postures during the user's movements,

• represent the movement by using a graphical representation given by the avatar.

In the calibration phase the user which wears the ULKG holds a set of position *θ*_*i *_(*i *= 1 ... *p*, where *p *is the number of positions to be recorded) and the sensor status **s**^**c**^_*i *_is acquired and stored in the *k *× *p *calibration matrix 



In the recognition phase, while the user moves the upper limb, the kinematic configurations are detected by acquiring the sensor outputs **s **and comparing them with the p columns of the calibration matrix. If the distance induced by the norm as defined in equation (7) between the actual sensor values and a column of the matrix is smaller than a certain threshold, the ULKG returns the position related to the selected column. In this application, it is not necessary that the entire space of the sensor values is mapped into the configuration space, so any other norm, instead of the one defined by equation(7) can be used. The system has also been tested by implementing the euclidean norm, and it has led the same results. When a posture is recognized, the visualization software performs an animation from the old position to the actual one. This transition is interpolated by using quaternions algebra: orientations acquired during the calibration in terms of Euler's angles are translated into unit quaternions and the movement from the old position *d *to the arrival one *a *are defined through the spherical linear interpolation algorithm [16]



which provides the interpolated quaternion *q*_*int *_at each time *t*. Moreover, the absence of singularities in unit quaternions permits the execution of each arbitrary trajectory in the configuration space. In other words, the possibility of executing and representing each movement allowed by the physical constraint is ensured.

### The ULKG as Posture Detector

According to the previous sections, the ULKG is able to record the sensor status in a finite number of positions in the configuration space. These data can be associated to corresponding positions to define a discrete map between subsets in the two spaces. An example of this map is the function which relates the centers of the clusters in the lattice introduced in section The UKLG Working Modes with the corresponding geometrical configuration. If the set of the points considered in the configuration space satisfy some particular requirements [7], this map can be extended by interpolation techniques to all the configuration space. A complete treatment of the requirements necessary to extend the function to all the configuration space is beyond the purpose of this paper. In [7] it is proved that the choice of a lattice having the same dimension of the space Θ ensures the possibility to extend the discrete map to a continuous one, *F *to all the space. Moreover a piecewise linear interpolation technique based on the decomposition of Θ into a lattice compounded by hypertetrahedra has been presented to construct *F *in the same work. The choice of the PL interpolation is due to the necessity to invert (or more generally, compute a pseudoinverse, *F*^†^, in case the dimensions of Θ and **S **do not match). PL functions are linear applications expressed by matrix, almost locally, and are invertible with low computational cost. If *F*^† ^is available and the set of configurations is coded by a parametrization, we know the position with a precision that depends on the interpolation used and the choice of the lattice used to compute the value corresponding to the sensor status of any acquisition. Moreover the procedure for the determination of the position consists only in the detection of the piece of *F*^† ^which holds for the particular sensor values *s *and multiplication *F*^† ^× *s*. The determined value for the position in the configuration space, can be continuously represented by the avatar, which in this case does not require interpolation techniques to represent an animation. A crucial point in the building of *F *is the choice of a parametrization for Θ. An additional subsidiary measurement system (constituted by a set of electrogoniometers produced by Biometrics Ltd.) has been employed to parametrize the configuration space Θ relating position to numerical values. The construction of *F *correspond to the identification of the parameters of the entire system, being defined by a field of matrices on Θ.

### The ULKG as a part of a post-stroke service

As mentioned in the introduction, the proposed technology is under testing in the field of post-stroke patients' rehabilitation. The main institution involved in the research and experimentation of the system to be employed in a medical environment is the S. Maugeri Foundation, in Pavia, Italy. This unit is responsible for the drawing up of a post-stroke rehabilitation protocol for hemiplegic patients according to the guideline contained in [17]. The most frequent damage in the adult stroke population concerns body district controlled by the brain areas depending on posterior and medial cerebral artery, causing plegia first and then spasticity to the upper and lower limb. More precisely, movement dysfunctions arise from a complex interaction among positive symptoms (spasticity, released flexor reflexes), negative symptoms (loss of dexterity and weakness) and changes in the physical properties of muscle tissues. These patients show clinical deficits that may include impairment of sensation, perception, cognition and motor control: together, these impairments contribute to functional limitations in mobility, posture maintenance, cares, comfort and many activities of daily living, such as to pick up a glass or to turn the pages of a book. Thus, the principal objective of rehabilitation in these patients is to improve daily functions. For our prototype, we chose to consider long term rehabilitation therapy of upper limb; in particular, we considered the shoulder and the arm. In this section we introduce the entire health care service including all the support structure of data management and communication required to improve the patients treatment both in the hospital and at home. The clinical pathway that a person affected by Stroke experiences after the event comprehends multiple healthcare environments, and depends also on the national healthcare system. In the following we refer to the Italian setting. The first step is admission in a unit for acute care for about 8–12 days. Then most of the patients, and particularly hemiplegic ones, are admitted to an Intensive Rehabilitation unit for about 30–45 days. Subsequently, if needed, patients are admitted to an Extensive Rehabilitation unit (in-patient unit where treatment lasts for no more than one-two hours a day) for about 30–40 days. Otherwise, they go home, or they enter the so called long-stay units, which host patients that, mainly for family reasons, cannot stay at home. During this intensive rehabilitation period, patients perform physical exercises with the help of physiotherapists, up to three hours each day. It is very important to continue such exercises after this period, even if with a lower intensity. According to the discharge conditions, physicians decide a personalised protocol: patients must repeat some exercises one or more times a day for a certain number of days, usually one-two months. These exercises are illustrated to the patient before discharge, but physicians could decide to update them later on, according to the patient's status modification. However, after discharge, several problems may arise, impairing the continuity of care:

• patients that go back to home, without an healthcare professional stimulating them, are poorly motivated to do regular exercise

• home caregivers may be not prepared adequately to give the intended support

• patients admitted to long-stay units or long term care units often worsen their psychological state, and this in turn decreases disposition to do exercise

• long-term care units and extensive rehabilitation settings often do not comply to evidence based rehabilitation protocols, and they have no link with the medical team that cared for the patient during the intensive rehabilitation period

We think that providing the patient with a virtual trainer for his rehabilitation could help to overcome these problems. In the following, the patient is intended to be at home, or in a long-stay unit, or in an extensive rehabilitation unit. The basic idea about this application is that when the patient logs on, the system prompts him with the current status of the rehabilitation protocol, and proposes the schedule of the day. The patient wears the sensorized garment and performs the exercise with the help of a movement tracker on the PC screen. At the end of the exercise, a global error measure is given to the patient in such a way that he can decide to repeat the task to improve his performance. Thus, the device facilitates the patient in performing in the correct manner the rehabilitation exercise. But, when a new technology is proposed, mainly in the outpatient care context, great attention must be devoted to the user interface. Technologically advanced devices may fail because of scarce usability or compliance. This is a crucial issue when dealing with elderly people, as in the case of the majority of post-stroke patients. Thus, the patient must be provided with a system that is as much easy to use as possible, to allow facing multiple problems through the same interface, without requiring an extensive learning effort. In our case, this means that the sensorized shirt must be not only a means for collecting data for further analysis, but it also must be integrated into a service able to:

• act as a patient-tailored support system, providing an immediate feedback about the patient's performance on a specific exercise, high-lighting, if any, the incorrect movements,

• show the patient's trend (i.e. improving, stationary, etc) in a given time interval, through easy-to-understand metaphors, such as a plant that grows up or that slows down,

• provide educational material, such as post-stroke rehabilitation guidelines, or movies illustrating the correct (and incorrect) movements for the specific patient's disability,

• allow communication between patient and health care providers.

From the health care provider side, it is important for the new service to be smoothly integrated into the clinical work-flow and take into account organizational issues. Thus, different functionalities are needed:

• providing an overview of patients enrolled in the rehabilitation treatment,

• following multiple patients in real-time,

• retrieving an exercise and send comments to the patient,

• allowing to send new exercise protocols to patients,

• maintaining the control of the service flow.

To support these functionalities, we developed a database, whose Entity-Relationship model lead to several tables that will store

• personal data of both patients and health care professionals,

• the objectives of the rehabilitation,

• the description, planning and execution of the exercises,

• the garment details,

• the messages between patients and hospital team.

From the communication infrastructure point of view, the system will be made by three main stations, located at different sites, and interconnected among them. The three sites, are

• the Patient Site, physically located near the patient, who wears the sensitive garments. The Patient Site computer is connected both with the Server Site, and with the electronics which interfaces to the garments.

• the Physician Site, from which the physician can monitor the patient's exercises. As mentioned above, the monitoring can happen both in real time (on-line) and on the stored sessions (off-line)

• the Server Site, where a firewall-protected central server hosts the database described above and all the necessary software to serve web pages dynamically generated to provide easy access to the system.

## Results

All the patient management system, work-flow and health-care service described in the previous section are currently under test for a clinical validation and no results on the matter is reported in the following. In the near future, we plan to collect all the achievements deriving from the clinical experimentation of the integration of ULKG in the health-care service. Here, only technical results deriving from the prototype validation, are reported. In our laboratories, the ULKG has been submitted to a series of trials in order to check the real capability of the instrument to recognize and detect gestures, postures and movements.

### The ULKG Performances as Posture and Movements Recorder

**Figure 5:**
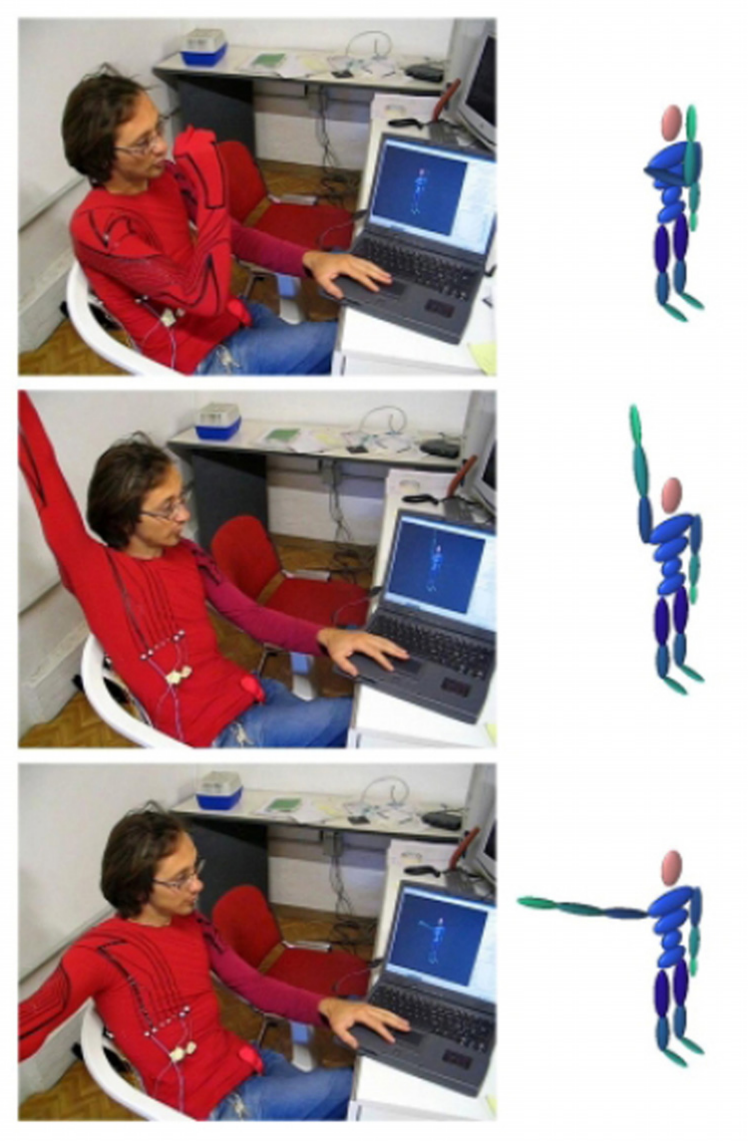
Posture recognition trials performed by the user and represented by the avatar.

The first basic working mode which has been tested is the ULKG functionality as posture recorder. The system has been used to record postures for the upper limb which have been related to the corresponding configurations in the model represented by the avatar. After having stored all the data concerning fifty different postures in the upper limb workspace, the same ones have been held again several times. The output of the ULKG was visualized on a computer screen, where the avatar replicated the subject's posture (Figure 5). The graphical representations has been performed by the avatar according the quaternion interpolation algorithm presented in section The ULKG as Posture and Movements Recorder with good animation quality. The system recognized 100% of the postures recorded, and no further re-calibration was thought to be necessary even if the ULKG had been removed and re-worn. Postures used to test the prototype included generic positions typically seen in the workspace. This trial tested both the hardware of the prototype and the clusterization and reconstruction algorithms described in section The ULKG Working Modes.

### The Performances ULKG as Posture Detector

**Figure 6:**
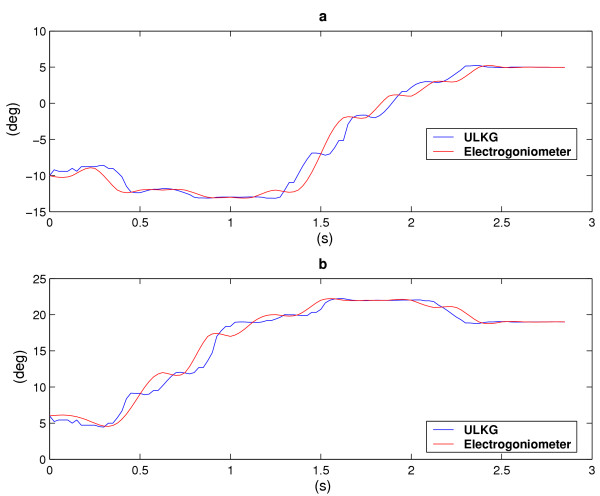
Flexion (a) and abduction (b) angles of the wrist versus time. The red line is the goniometer output, while the blue one represents the ULKG response.

**Figure 7:**
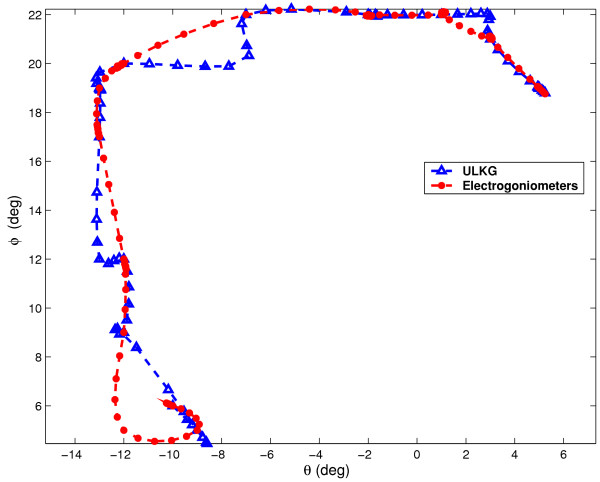
Composition of the flexion angle (in abscissa) and abduction angle (in ordinates) of the wrist. The red line is the goniometer output, while the blue one represents the ULKG response.

According to section The ULKG as Posture Detector the prototype was tested through several trials to evaluate its performances in dynamic working conditions and during the detection of unknown posture. The main powerful demonstration gathered from these trials is that the ULKG is able to reconstruct postures never recorded or held before. In each trial the ULKG was worn by a subject and a set of electrogoniometers was positioned on the user. The goniometers were adequate to detect only flexion-extension (and adduction-abduction) executed by the joints under study and they were used only to have a description of the movements performed. Torsions are not relivable by using this instrumentation. The theoretical resolution provided by the producer is 0.5 degree. No interactions between the ULKG and the goniometers were allowed. The subject was invited to perform a set of movements which involve the gleno-humeral joint, the elbow and the wrist, like flexions-extensions, abductions-adductions and circling of the body segments. Signals deriving from the ULKG and from the set of goniometers were simultaneously acquired. The outputs of the ULKG was processed according to section The ULKG as Posture Detector and the results obtained in terms of angles were compared with the goniometers output. Data obtained from these experiments are showed in two different presentation. The first one is a classical representation of the angle values versus time. In the plots, both the ULKG output and the values presented by the goniometers are shown and compared. In the other representation, we have considered some planes contained in the configuration space 0 and we have plotted the trajectories performed by some joints on them, both for goniometers and for the ULKG. This presentation is very powerful to detect divergences between the two responses. In Figures 6, 7 the analysis of a wrist rotation is reported. Figures 6a and 6b show the flexion and abduction angles (which compound the movement) versus time. The red line is the goniometer output, while the blue one represents the ULKG response. Figure 7 composes the two angle evolutions in a trajectory which meaningfully explain the motion. Flexion is reported on the abscissa axis, while the abduction is reported on the axis of ordinates. The colors used for goniometers and ULKG are the same of Figure 6.

**Figure 8:**
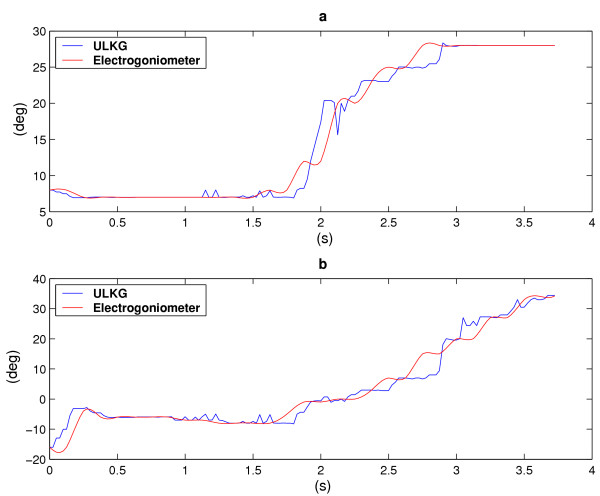
Extension (a) and flexion (b) angles versus time of the shoulder. The red line is the goniometer output, while the blue one represents the ULKG response.

**Figure 9:**
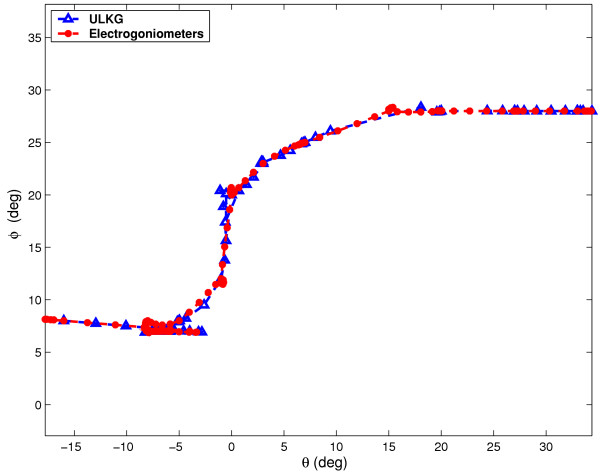
Composition of the flexion angle (in abscissa) and extension angle (in ordinates) of the shoulder. The red line is the goniometer output, while the blue one represents the ULKG response.

The same scheme has been adopted to report a movement for the shoulder in Figures 8, 9. Extension is reported in Figure 8a (versus time) and on the y-axis of the Figure 9. Conversely, Figure 8b and the x-axis of Figure 9 represent the evolution of the shoulder flexion.

**Figure 10:**
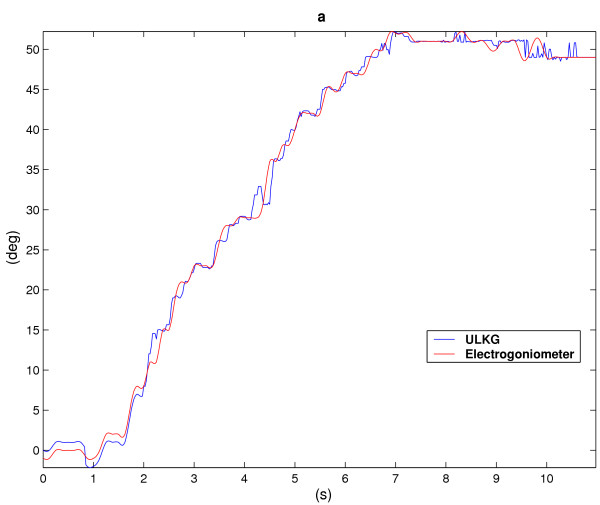
Flexion angle of the elbow. The red line is the goniometer output, while the blue one represents the ULKG response.

Finally, an elbow flexion is shown in Figures 10. Both shoulder rotation and elbow pronationsupination have performed qualitative results in terms of sensor signal trends but these responses have not yet been analyzed because the electrogoniometers we used are not capable to detect such responses and an identification of the ULKG along this movement direction has not been possible.

The results are similar to the ones demonstrated in [7]. The only difference resides in the statical character of the trials in our previous work, while in this case movements are described by the ULKG output. Two kinds of divergence between the ULKG behavior and the goniometer responses are pointed out by the introduced diagrams. The first error we can note is a real divergence between the information deriving from the two measurement systems. Estimated trajectories differ for a certain quantity and this phenomenon can be observed both in the plot showing "angle versus time" and in the one showing trajectories. It is clearly pointed out in Figure 7 in the range [-12°, -8°] for flexion angle and [4°, 8°] for abduction angle. Evaluated in cartesian norm, the error estimated is anyway smaller than 5 per cent, if compared with the dimension of the entire workspace. The other artifact we can note is a difference between signals in the "angle versus time" plots, that is not detectable by watching the other representation. This phenomenon is due to a lack of synchronization between the two measurement system and it is manifest in Figure 8a and 8b in the [0, 0.5] second range, without corresponding to an effective difference in terms of trajectory, as demonstrated in Figure 9. The two systems lead to the same results at different time. A refinement of the movement detection algorithm may avoid these two errors and will be studied in the future.

## Conclusion

In this manuscript, an upper limb kinesthetic garment for gesture, posture and movement detection has been presented. The main advantage introduced by this prototype is the possibility to wear it for long periods of time thus allowing clinicians to monitor patients without causing any discomfort. Several issues, deriving from the employment of the new technology which has allowed the realization of the unobtrusive device, are addressed. In particular a modeling for the physical behavior of the sensor employed was proposed. An algorithm of signal analysis derived from the model was implemented to allow the use of conductive elastomers as sensors. Moreover both the implementation of the sensing prototype and its performances as posture recorder and posture detector were introduced. We used particular care in explaining all the algorithms necessary to reconstruct or estimate posture and movement. We discussed both the application of a the classical biomechanical methodology as well as some innovative techniques whose development we deemed necessary to ensure good results in the garment employment. An application of the upper limb kinesthetic garment as useful instrumentation in post-stroke rehabilitation was described, together with a complete description of the clinical service where the garment is integrated. Finally, results on the performances of the sensing system were reported.
